# Cytomegalovirus infection can mimic genetic nephrotic syndrome: a case report

**DOI:** 10.1186/s12882-015-0152-z

**Published:** 2015-09-22

**Authors:** Julien Hogan, Marc Fila, Véronique Baudouin, Michel Peuchmaur, Georges Deschênes, Olivier Niel

**Affiliations:** Pediatric Nephrology Department, Robert Debré Hospital, 48 boulevard Sérurier, 75019 Paris, France; Inserm U1163, Molecular Pathways of Hereditary Kidney Diseases, Imagine Institute, 24, boulevard du Montparnasse, 75015 Paris, France; Cytology and Pathology Department, Robert Debré Hospital, 48 boulevard Sérurier, 75019 Paris, France

**Keywords:** Cytomegalovirus, Kidney, Nephrotic syndrome, Proteinuria

## Abstract

**Background:**

Nephrotic syndrome is a relatively rare but serious condition in children. Infantile nephrotic syndrome often has a genetic origin; the treatment is then symptomatic, with a poor prognosis, and a rapid evolution to chronic kidney disease. However, non-genetic infantile nephrotic syndrome has been identified. Here we report for the first time in a child a nephrotic syndrome as the sole clinical expression of a cytomegalovirus infection.

**Case presentation:**

The patient was 5 months old when he presented with a nephrotic syndrome. An exhaustive genetic testing was conducted and came back negative. A viral work-up only showed a positive cytomegalovirus PCR. Antiviral treatment lead to a complete remission of the nephrotic syndrome, with no requirement for steroid therapy. Renal function remained normal throughout follow-up.

**Conclusion:**

Nephrotic syndrome should always be carefully investigated in children. This observation reinforces the connection between viral infections and pediatric nephrotic syndrome, sparking more controversy about an infectious origin to childhood nephrotic disease.

## Background

In the first year of life, 66 % of the patients suffering from hypoalbuminemia and severe proteinuria are diagnosed with genetic nephrotic syndrome (GNS) [1]. Many mutations can be responsible for GNS. NPHS1, encoding nephrin, is involved in the Finnish type of GNS [2]; 2 main variants are characterized, Fin-major (p.L41fsX91) which represents 78 % of the described NPHS1 mutations in Finland, and Fin-minor (p.R1109X) [3]. Podocin, a protein which is encoded by the NPHS2 gene, has also been reported in GNS cases. Noteworthy, immunosuppressive treatments are often inefficient; the prognosis of the disease is poor, and renal transplantation is often necessary, usually with no relapse.

However, non-genetic congenital or infantile nephrotic syndromes have been identified. They have to be diagnosed early, since some of them can be treated in a specific manner, and cured [4].

## Case presentation

The patient was 5 months old when he presented with acute gastroenteritis; he had no previous medical history, except for 3 episodes of bronchiolitis. He had a twin brother, from a dichorial diamniotic pregnancy; the twin brother was in good health. The parents were Caucasian, and not consanguineous. There was no significant family medical history. Clinically, mild edema was noted on the face and lower limbs. Serum creatinine was normal (35 μmol/l, normal range 25–45 μmol/l), serum urea was also normal (5.4 mmol/l, normal range 4–7 mmol/l). Total serum protein came back low, measured at 35 g/l (normal range 60–75 g/l), and albuminemia was also low, at 10.3 g/l (normal values 35–45 g/l) as shown in Table 1. Proteinuria was elevated, at 5.1 g/l, leading to a proteinuria/creatinuria ratio of 7.83 g/mmol. A renal ultrasound showed a right kidney measured at 65 × 33 × 30 mm, and a left kidney measured at 71 × 35 × 28 mm; the Doppler analysis was normal. Kidney size remained stable throughout follow-up, above average. There was no sign of blockage, no stone, no tumor. A renal biopsy was performed, analyzing 13 glomeruli; it showed a slight mesangial cell hypertrophy with no diffuse mesangial sclerosis; non-specific tubular lesions were present, along with interstitial edema. No viral cytopathic inclusions could be seen. Electron microscopy was not performed. A viral work-up came back negative for hepatitis A, B and C; syphilis and HIV serologies were also negative. A genetic testing showed no mutation in NPHS1, NPHS2 nor WT1 genes. Hip and knee radiographs were normal, eliminating a nail patella syndrome. At this stage, angiotensin converting enzyme inhibitor therapy was initiated; captopril was used, started at 3 mg/d, and increased to 6 mg/d after 15 days. Albumin perfusions were performed initially, at a dose of 1 g/kg/d, each day, for 16 days.

**Table 1:** Evolution of albuminemia, total serum protein and proteinuria in a patient with cytomegalovirus-induced nephrotic syndrome

Time (d)	Event	Albuminemia (g/l)	Total serum protein (g/l)	Proteinuria (g/l)	CMV viraemia (copies/ml)
0		10.3	35	5.1	123000
7	Albumin perfusions	24	52	4.8	
15	ACE therapy	25	54	4.1	
30	Ganciclovir perfusions	30.2	60.2	0.15	12000
45	Valganciclovir therapy	34	62	0.16	

Epstein-Barr virus PCR test was negative; cytomegalovirus PCR test was positive, showing more than 123,000 copies/ml. Cerebral imaging showed no significant brain lesion; An EEG was performed and came back normal. Ophthalmoscopy was also normal. Ganciclovir was immediately started and administered for 15 days; it was then switched to valganciclovir for another 15-day period. By the end of the antiviral therapy, proteinuria had decreased to 0.15 g/l; albuminemia and total serum protein levels were back to normal values. Captopril therapy could successfully be discontinued. No relapse had occurred in 30 months.

## Discussion–Conclusion

Here we describe an infantile nephrotic syndrome, in relation with a CMV infection. Interestingly, the nephrotic syndrome resolved when proper antiviral therapy was initiated, supporting a causal relationship between infantile nephrotic syndrome and cytomegalovirus infection. Noteworthy, another case of cytomegalovirus related nephrotic syndrome has been described [5]; but, as opposed to this report, extra-renal symptoms were predominant over the renal presentation. On the contrary, the clinical presentation reported here was misleading, since only a slight digestive symptomatology was in favor of a systemic etiology. This observation reinforces the connection between viral infections and nephrotic syndrome in children [6], sparking more controversy about an infectious origin to childhood nephrotic disease.

### Consent

Informed consent was obtained from the patient’s parents for publication of this case report.

## Abbreviations

CMV: Cytomegalovirus
GNS: Genetic nephrotic syndrome
NPHS1: Nephrin gene
NPHS2: Podocin gene
WT1: Wilm’s tumor 1 gene
